# Clock Glitch Fault Attacks on Deep Neural Networks and Their Countermeasures

**DOI:** 10.3390/s25092793

**Published:** 2025-04-29

**Authors:** Sangwon Lee, Suhyung Kim, Seongwoo Hong, Jaecheol Ha

**Affiliations:** 1 Department of Information Security, Hoseo University, Asan-Si 31499, Republic of Korea; sangone9629@naver.com (S.L.); aa5551107@naver.com (S.K.); 2 EV R&D Team, Coontec Co., Ltd., Seongnam-Si 13449, Republic of Korea; hshsw5660@gmail.com

**Keywords:** sensor network, deep neural network, image classification, hardware security, fault injection attack

## Abstract

Recently, deep neural networks (DNNs) have been widely used in various fields, such as autonomous vehicles and smart homes. Since these DNNs can be directly implemented on edge devices, they offer advantages such as real-time processing in low-power and low-bandwidth environments. However, the deployment of DNNs in embedded systems, including edge devices, exposes them to threats such as fault injection attacks. This paper introduces a method of inducing misclassification using clock glitch fault attacks in devices where DNN models are executed. As a result of experiments on a microcontroller with a DNN implemented for two types of image classification (multi-class and binary classification using MNIST, CIFAR-10, and Kaggle datasets), we show that clock glitch fault attacks can lead—with a high probability—to the occurrence of serious misclassifications. Furthermore, we propose countermeasures to defeat the glitch attacks on each Softmax function and Sigmoid function at the algorithm level, and we confirm that these methods can effectively prevent misclassification incidents.

## 1. Introduction

Recently, deep learning has been applied in various real-life applications, such as autonomous vehicles, smart homes, and IoT applications. However, these deep neural network (DNN) implementations are easily exposed to threats from malicious attackers when operating on systems that are far away from their administrators, such as edge devices. For instance, in adversarial attacks, an attacker deliberately perturbs the input data to induce misclassification in DNNs designed for image recognition [1,2,3]. Additionally, they can also induce misclassifications by physically accessing the DNN computations performed on edge devices and injecting faults into the internal computational processes [4,5,6,7,8]. In practice, fault injection attacks allow adversaries to compromise security mechanisms in edge devices in smart homes and bypass authentication processes [9]. They also pose a serious threat to applications such as object detection in autonomous vehicles, as they can cause operational flaws [10].

Many of the fault injection attacks developed thus far, including glitches [11,12,13], lasers [14,15], electromagnetic (EM) pulses [16,17], and other methods compromising device operation, have been used to introduce faults into encryption systems. Here, the glitch fault injection attack is conducted with direct contact to the target device, making it a highly accurate and relatively low-cost technique. Comparatively, fault attacks using lasers or EM pulses are non-contact methods, so the accuracy of the attack is relatively low and the cost of building an attack environment is high. Moreover, they require chip decapping for accurate fault injection, which poses challenges in practical applications. In voltage glitch attacks, direct access to the power supply line of the target device is required, and the device may stop operating due to power supply instability. In contrast, clock glitch attacks, in which the edge timing of the main clock is disrupted, can be carried out more easily due to simpler synchronization and a stable power supply.

Fault injection attacks enable adversaries to analyze faulty outputs and extract secret information stored inside devices [18,19,20,21]. Recently, various attacks have been established with the ability to inject faults in DNNs, thus inducing misclassifications [10,22,23,24,25,26,27]. Liu et al. proposed a white-box attack on DNNs that is based on software simulation [22]. However, it did not provide practical results in real implementation scenarios. Zhao et al. proposed a framework for injecting stealth faults into selected inputs while maintaining the overall test accuracy [23]. Practical research by Bereir et al. used laser-injection techniques on embedded devices to conduct fault injection attacks; this research was conducted to demonstrate the possibility of inducing misclassification through the injection of faults into the hidden layers of DNNs [10,24]. Alam et al. proposed remote fault attacks that utilized the vulnerabilities in the RAM of FPGAs, demonstrating an ability to induce timing faults and bit flips [25]. Fukuda et al. evaluated attack performance from faults caused by injecting clock glitches into the Softmax function of a DNN output layer, successfully misclassifying input images processed on an 8-bit microcontroller [26]. Recently, Ordoñez et al. conducted an experiment in which faults were injected into the fully connected (FC) layer of a black-box DNN model. Their results indicate that up to 93% of inputs were misclassified as attacker-specified classes, demonstrating the method’s effectiveness [27]. In another study, Das et al. proposed a method for inducing bit flips in large language models (LLMs) using software-induced faults such as Rowhammer attacks to undermine the integrity and performance of the models [28].

In this study, we experimentally demonstrate that a clock glitch fault attack on a DNN used in an edge device can lead to misclassifications. We injected a clock glitch capable of inducing misclassifications with high probability, using simple tools and with low experimental cost. Since these glitch attacks are more effective than attacks on other activation functions in the hidden layers, they primarily target two functions in the last layer of the DNN: the Softmax function for multi-class classification and the Sigmoid function for binary classification.

The experiments launch clock glitch fault attacks on devices running deep learning models. Specifically, the attacks target the activation functions used in the output layers of multi-class and binary classification models (namely Softmax and Sigmoid) by inducing instruction skipping to cause misclassifications. In addition, considering the realistic scenarios of glitch fault injection attacks, we use an external trigger-generation method to determine the exact timing on the target device. This method uses the Sum of Absolute Differences (SAD) algorithm [29], a pattern-matching technique between the real incoming waveform and a reference waveform. Here, the reference waveform is a type of pre-enrolled side channel information, such as power consumption.

The major contributions of our paper are as follows:

- We launch fault injection attacks on the output layer activation functions of two models: a multi-class classification model trained on the MNIST (10 digits) and CIFAR-10 datasets, and a binary classification model trained on the MNIST (two digits) and Kaggle (Dogs vs. Cats) datasets.
- Considering the practical feasibility of fault injection attacks, we conducted experiments by generating glitches outside the target device. Furthermore, we used a waveform pattern-matching algorithm based on the power consumption waveform at the attack point to determine the timing for the clock glitch injection.
- We implemented several classification DNN models on the target chip ARM Cortex-M4 STM32F303, and confirmed that these models are vulnerable to fault attacks.
- Considering that only a negligible computational overhead should be added, the proposed countermeasures effectively mitigate the attacks on Softmax and Sigmoid functions through additional detection processes at the algorithmic level.

Compared to previous similar studies in the literature [22,24,26], this work confirms that fault injection attacks can be extended to various applications of deep learning models. In particular, we conducted practical experiments on both binary and multi-class classification models. In addition, we emphasize that software countermeasures at the algorithm level are very effective against fault injection attacks on Softmax and Sigmoid functions.

This paper is structured as follows. Section 2 describes the DNN structure and the fault injection attacks. In Section 3, we analyze the Softmax and Sigmoid functions that are the targets of the attack and observe possible attack points. Section 4 describes the experimental settings for the microcontroller; additionally, the results from the clock glitch fault attacks on a DNN are presented. Section 5 explains the principles of our countermeasure and presents experimental results when applying the countermeasure on the targeted board. Section 6 concludes the paper.

## 2. Backgrounds and Methods on Fault Injection Attacks

### 2.1. The DNN Structure

**Figure 1:**
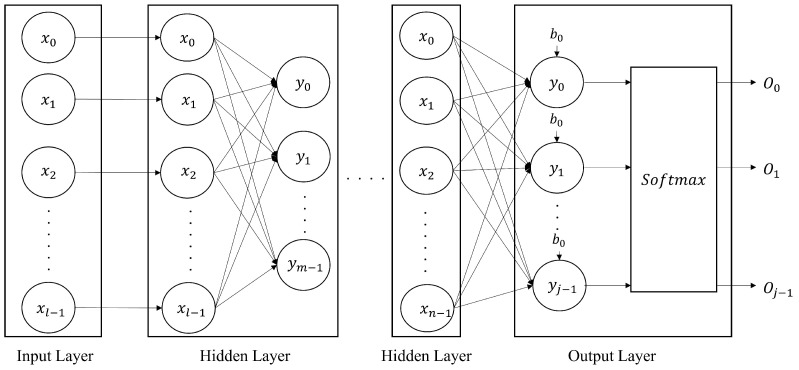
Structure of the MLP model.

DNNs are artificial neural networks with multiple hidden layers that learn complex patterns in data. They are widely used in areas such as image recognition and natural language processing because they can learn complex and nonlinear data patterns using a network of neurons. The basic form of the DNN, a multilayer perceptron (MLP), consists of an input layer, hidden layers, and an output layer. The structure of the MLP model is shown in Figure 1.

Activation functions used in DNNs are functions that transform the sum of input signals at each neuron in the neural network in a nonlinear manner. These activation functions are essential in enabling the network to learn and classify complex data patterns. The representative activation functions include Sigmoid, tanh, ReLU, and Softmax. Softmax is commonly used in the output layer of DNN models that process multiple classes by transforming output values into probabilities for each class. That is, each output represents the probability of belonging to a certain class.

The formula for calculating the input value, $y_{i}$, of the *i*th neuron in the Softmax function is shown in Equation (1). Here, *J* represents the number of classes, indicating the number of neurons in the output layer.

(1) $$Softmax\left(y_{i}\right)=\frac{exp(y_{i})}{\Sigma_{k=0}^{J-1}exp\left(y_{k}\right)}$$

Since the Softmax function normalizes the classification probability of the image, it is characterized by the sum of each output: this being 1. Based on these special properties, classification models can interpret the Softmax output as the predicted probability for each class. This study focuses on the Softmax function commonly used in the output layer of most classification models. The meaningful injection of malicious faults into the final output nodes of an image-classification DNN can lead to misclassification.

The Sigmoid function is a widely used activation function that transforms input values into the range of 0 to 1. This characteristic makes it particularly suitable for binary classification problems, where the model needs to output probabilities. By compressing its input into this limited range, the Sigmoid function ensures that the output can be interpreted as a likelihood score, providing a meaningful measure of confidence for classification tasks. The mathematical expression of the Sigmoid function for a given input, *x*, is as follows:

(2) $$Sigmoid\left(x\right)=\frac{1}{1+e^{-x}}$$

The Sigmoid function is commonly used in the output layer of binary classification models, where the output of this function represents the probability that the input belongs to a specific class. Typically, if the output value is greater than 0.5, the input is class 1; otherwise, it is class 0.

### 2.2. Fault Injections

In a fault injection attack, a fault that is designed to induce a malfunction is inserted into a device. These attacks can be used to compromise the stability of a system or to extract secret data. Fault injection attacks can be conducted through various methods, including glitches in the clock or the voltage of the target device. In this study, we conduct fault injection attacks based on the clock, which are low-cost and high-performance methods compared to other attacks.

**Figure 2:**
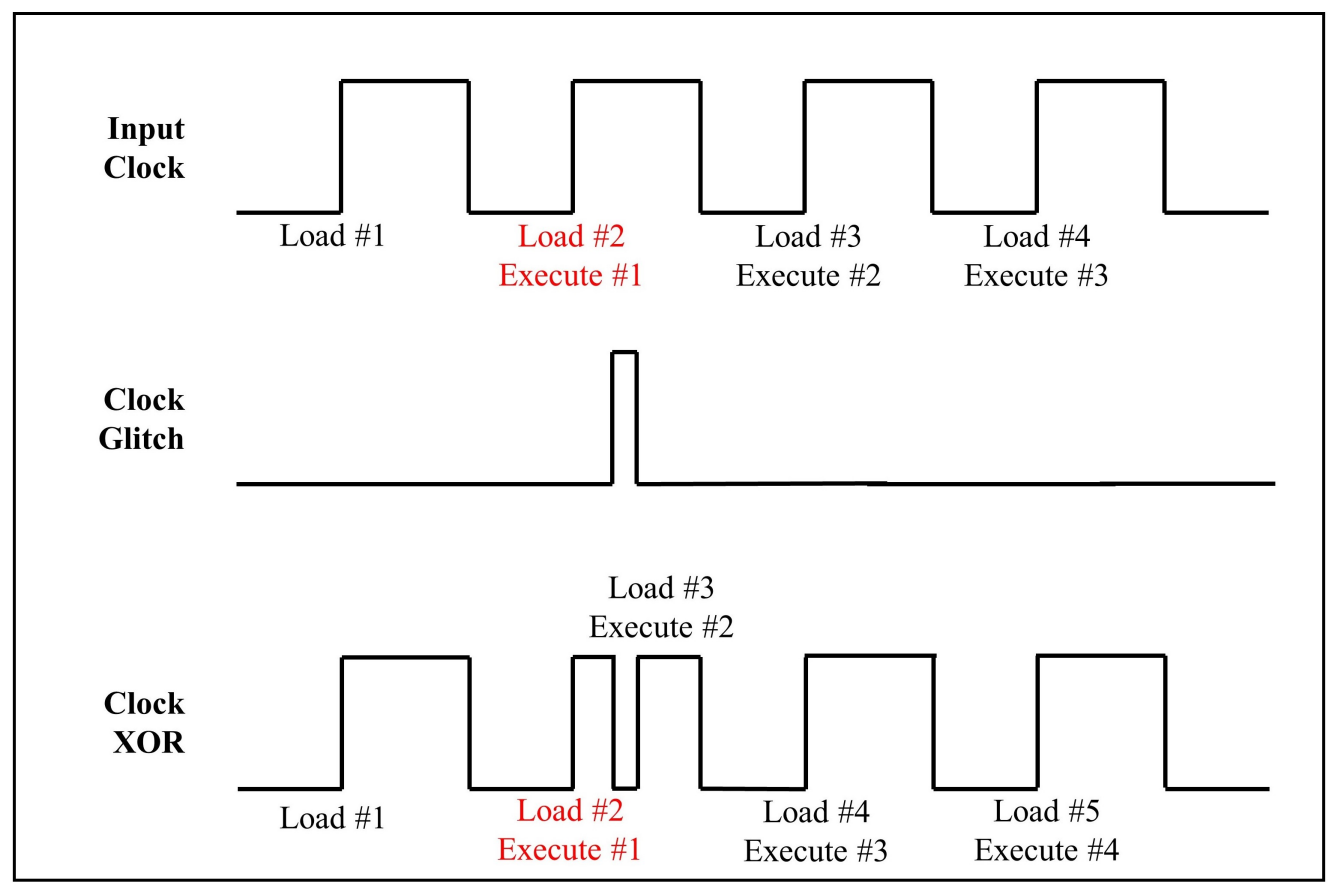
The effect of a clock glitch on pipeline instructions (Load instruction 2 and execute instruction 1 at a second clock).

In general, the clock controls the timing of operations in a computing device. If abnormal behavior occurs in this clock signal, instructions may not work properly. Furthermore, these faults may lead to an abnormal termination or incomplete use of memory. A malicious attacker can induce abnormal operations by injecting clock glitches while the target device is running. Figure 2 illustrates the effect of a clock glitch on pipeline instructions.

**Figure 3:**
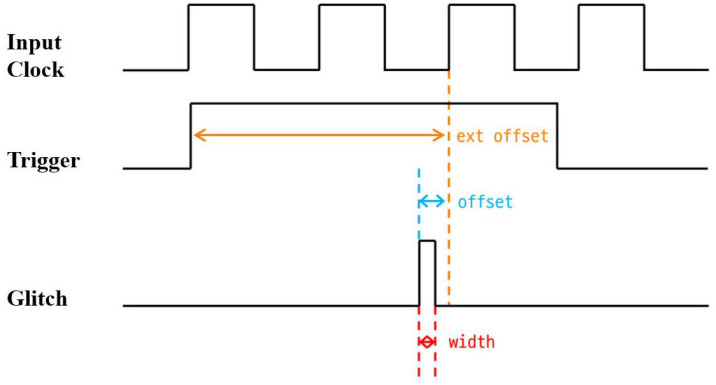
Clock glitch parameters (Orange: ext_offset, Blue: offset, Red: width).

In a clock glitch fault attack, the selection of several parameters that characterize the glitch signal is an important factor in determining whether the attack is successful. There are three glitch parameters: width, which specifies the duration of the glitch, offset, which determines when the glitch occurs within a clock cycle, and ext_offset, which defines the delay from when the trigger is detected until the glitch is injected. These parameters for a clock glitch attack are illustrated in Figure 3. They may need to be fine-tuned depending on the clock time and characteristics of the device.

Typically, a high-performance FPGA circuit is embedded in the controller device to generate clock glitches. This device creates multiple phase-shifted copies of the original clock signal, thus producing multiple glitch streams. The controller then generates a clock glitch that is delayed relative to an external trigger signal and has variable width and offset characteristics.

To perform a fault injection attack, the adversary needs a trigger mechanism to determine the timing of the fault injection. The prevalent method in academic works is to generate a trigger signal from the target device. However, this approach is not possible in realistic scenarios. A more realistic alternative is a pattern-based triggering mechanism that consists of two stages. First, an adversary selects a suitable reference signal from a window of interest in the side channel signal. Second, a pattern-matching algorithm detects a similar waveform from an incoming signal.

There are various pattern-matching algorithms, but a SAD algorithm is adopted in this paper. This algorithm performs sample-wise subtraction between reference and incoming signals, and takes the sum of the absolute value of the difference, which is calculated as follows:

(3) $$SAD\left(k\right)=\sum_{m=1}^{N}\;\;\left|g\left(m\right)-h\left(m+k\right)\right|$$

In Equation (3), *g* is a fixed reference signal from samples of length *N*, and *h* is a continuous incoming one. A difference value for the two signals is computed at a certain time shift, *k*. Finally, if the signal score SAD(k) is less than or equal to the threshold, the reference pattern is determined to match the incoming waveform.

## 3. The Fault Injection Model

In this paper, we conduct clock glitch fault attacks targeting the activation functions in the output layer of both multi-class and binary classification models. The objective of these attacks is to maliciously induce incorrect classifications for the given input images. In our experiments, it is important to determine vulnerable timing points where misclassification may occur in the DNN classification model, and then generate triggers at those points using the SAD pattern-matching algorithm.

Algorithm 1—which is based on Equation (1)—shows the operational flow of the Softmax function, which is implemented in a microcontroller. In this algorithm, the output array *O* is first initialized to 0. In the following “For” loop, an exponential operation is performed on an input value, $y_{k}$, stored in array *O* at index *k*. Then, the values in $O_{k}$ are summed and stored in the variable sum. In the second “For” loop, $O_{k}$ is divided by sum. Consequently, the output array *O* holds values that are converted to a probability distribution from the value of each neuron in the *y* array.
**Algorithm 1** The Softmax function**Require:** 
$y(y\in \left\{y_{0},\ldots ,y_{J-1}\right\})$
**Ensure:** 
$O(O\in \left\{O_{0},\ldots ,O_{J-1}\right\})$
  1:**for** (k=0;k<J;k++) **do**  2:    $O_{k}=0$  3:**end for**  4:sum=0  5:**for** (k=0;k<J;k++) **do**      // Attack point on loop counter  6:    $O_{k}=exp\left(y_{k}\right)$  7:    $sum=sum+O_{k}$  8:**end for**  9:**for** (k=0;k<J;k++) **do**10:    $O_{k}=O_{k}/sum$11:**end for**


In multi-classification models, the probability of successful misclassification increases when fault injection is performed in the final output layer rather than in the hidden layer. In addition, we targeted the weak point where the iterative process is executed because it is more effective to skip the “For” loop statement in the algorithm than to change the internal register value by fault injection.

Here, glitches are inserted into the first “For” loop: lines 5–7 of the Softmax algorithm. The precise timing of this is as follows: the point after the operation k=0 in the loop has completed and before *k* becomes 1 during the first “For” loop. At this point, $O_{0}$ contains the correct values, but all subsequent values in the *O* array remain at zero. Additionally, only the value of $O_{0}$ is stored in the variable sum. Therefore, when the second “For” loop is executed, $O_{0}$ is 1, while the remaining classes have a value of 0.

**Figure 4:**
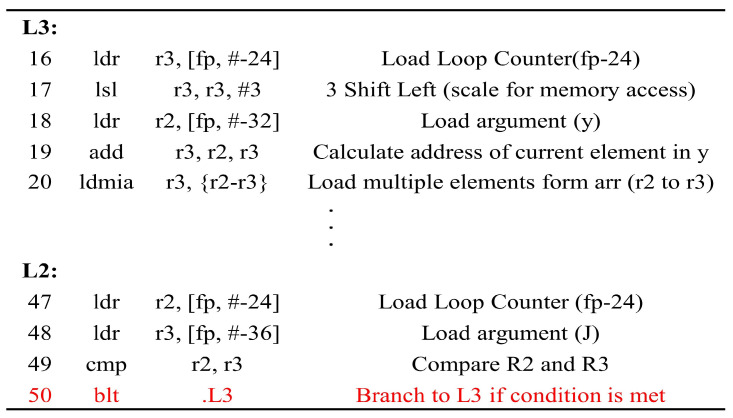
Assembly list of the Softmax function implemented on ARM Cortex-M4 STM32F303 microcontroller (Red line: statement for conditional branch).

The assembly list of the Softmax function implemented on ARM Cortex-M4 STM32F303 microcontroller, compiled from lines 5–7 of Algorithm 1, is shown in Figure 4. Label L3 represents the section performing the operations within the loop. Label L2 corresponds to the portion verifying the conditional branch; if *k* is less than *J*, then branching to L3 occurs. The injected glitch causes line 50 of the assembly list to be skipped, and it prematurely terminates the first “For” loop.

**Figure 5:**
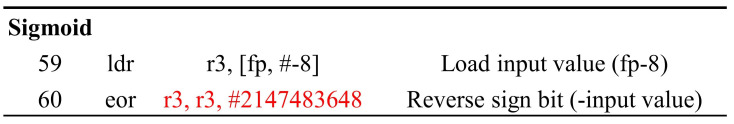
Assembly list of the Sigmoid function implemented on the ARM Cortex-M4 STM32F303 microcontroller (Red line: statement for reversing sign bit).

The Sigmoid function used in the output layer of the binary classification model is described in Algorithm 2, in which the output is expressed as a single value. If a fault is induced into this output function, it can result in significant misclassification rates. The assembly instructions for the Sigmoid function implemented on the microcontroller used in the experiments are shown in Figure 5.
**Algorithm 2** The Sigmoid function.**Require:** *x* **Ensure:** *y*1:y=1.0/(1.0+exp(−x))      // Attack point on input2:return  *y*

Among these instructions, we specifically target line 60 (which is responsible for flipping the sign of the input value) by applying a clock glitch to skip its execution. As a result, the relationship between faulty output $y^{\prime }$ and correct output *y* is shown in Equation (4):

(4) $$y^{\prime }=1-y$$

This means that if the Sigmoid output, representing the probability of a specific class, is inverted, all predictions in a binary classification model will be completely reversed.

## 4. Clock Glitch Fault Attacks on a DNN

### 4.1. Experimental Setup

The first target DNN model for fault injection attacks is MLP, which can classify digit images from the MNIST dataset [30]. Multi-class classification is the process of predicting input digit images ranging from 0 to 9 into 10 classes. In addition, we performed an experiment on a binary classification model that classifies images of 0 and 1. The MNIST dataset used in our experiment consisted of 60,000 samples for training and 10,000 samples for testing. In addition, the MNIST dataset used in binary classification consisted of 10,000 samples for training and 1000 samples for testing. Table 1 shows the MLP model structure for classifying the MNIST dataset used in our experiments. The input layer uses 784 neurons to receive images, and the output layer consists of 10 neurons producing the final result through the Softmax function. In a binary classification model, the output layer has a neuron and uses the Sigmoid function for the final output.

**Figure 6:**
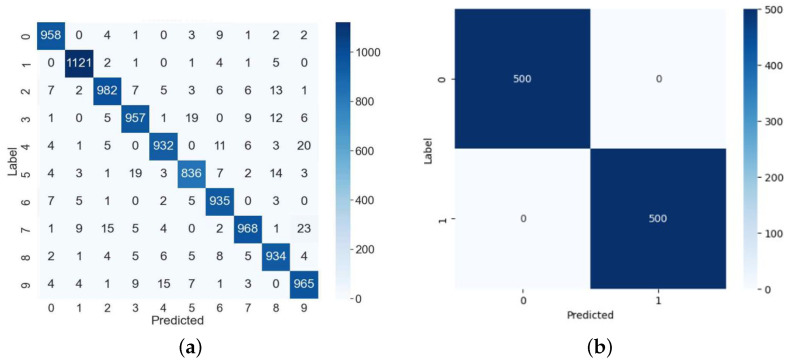
MNIST dataset classifications under normal MLP operation. (**a**) Multi-classification. (**b**) Binary classification.

Figure 6 shows classification of the MNIST digits by a normal MLP model that was not subjected to a fault injection attack. These results are classifications that occurred from using a dataset comprising 10,000 MNIST images for multi-class classification and 1000 images for binary classification. As shown in the figure, we can confirm the classifications achieved precisions of 95.88% and 100%, respectively.

In addition, when evaluating accuracy using a larger test set for both multi-class and binary classification, the difference in performance did not exceed ±0.08%, indicating that the size of the test dataset was adequate for performance analysis and that fault injection attacks initiated with test sets of this size yield reliable results.

**Figure 7:**
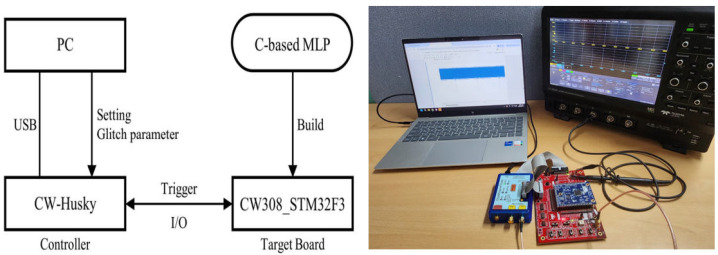
Setup and interface between experimental devices.

To evaluate the performance of our fault injection attacks, practical experiments were conducted using the ChipWhisperer-Husky platform from NewAE. Additionally, settings for the glitch fault injection attacks included the CW308 UFO Board, which is equipped with a Cortex-M4 STM32F303 microcontroller manufactured by ARM. The experiment setup and the interface connection between devices are shown in Figure 7. The PC was connected to the CW–Husky controller via a USB connection to adjust the environment parameters in the experiments, input data into the target board, and monitor the experimental results. The target DNN models were implemented in C and uploaded to the tested device using the ARM-GCC compiler.

Next, we determined the optimized width, offset, and ext_offset glitch parameters to identify the precise fault injection location when the trigger would occur in the CW-Husky. Once these parameters were determined, the target board was operated, and the SAD pattern-matching algorithm was used to locate the triggering event. Once the trigger occurred, a glitch fault was injected after the ext_offset time. Depending on the nature of the injected fault, the target DNN model may operate normally (in cases without fault injection), misclassify, or cause a malfunction in the target board.

### 4.2. The Results of Clock Glitch Fault Injection

In this study, we identified the optimal attack point by adjusting the parameter values offset and width used to generate the glitch, and identified the location for the fault injection to find the configuration with the highest success rate. The width and offset of the clock glitch generated using the ChipWhisperer-Husky were closely related to the clock of the target chip, STM32F303 (7.37 MHz), and the clock of the FPGA’s voltage-controlled oscillator (VCO), which operated at 604.35 MHz and was embedded in the controller. In this setup, the phase-shift step of a glitch was set to a maximum of 4592, calculated as (604.35 MHz/7.37 MHz) × 56 (a constant). During the experiment, the minimum and maximum bounds of this step were determined, and the characteristics of the clock glitch that successfully induced misclassification were identified.

In clock glitch attacks, core parameters such as width and offset must be precisely adjusted according to the clock frequency and the hardware architecture of the device. Therefore, the function of the controller initiating the attack may vary depending on the hardware structure, instruction processing method, and clock speed of the target device. In other words, various phase-shift steps and parameter settings are involved in the injection of clock glitches [26,31,32]. In particular, if the target device is an RISC-V or general-purpose microprocessor, the clock glitch attack may not be very effective as the hardware structure or operating environment is different. In this case, a high-performance specialized controller may be required, and non-contact fault injection tools and equipment may be utilized [33].

**Figure 8:**
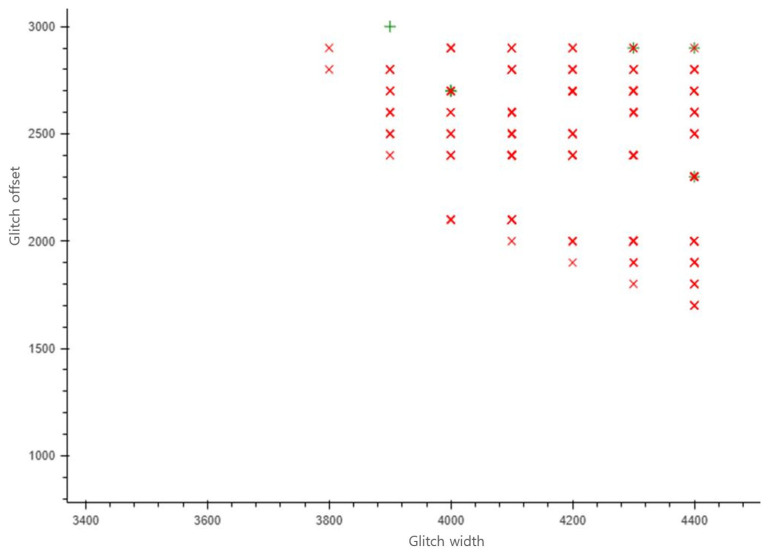
Parameter distribution map to find successful misclassification points.

The parameter distribution of the offset and width of clock glitches that lead to successful misclassifications is presented in Figure 8. In this figure, the x-axis represents the glitch width in phase-shift steps, while the y-axis indicates the offset at which the glitch begins, also in phase-shift steps. The selection of these two parameters is critical, as they play a crucial role in successfully skipping instructions. As shown in Figure 8, when the parameter value is indicated by a red cross, the device stopped responding, and when also indicated by a green plus sign, misclassification occurred. Notably, many misclassifications occurred when the width was between 3800 and 4400 and the offset was between 1700 and 3000. Since the location where the fault is injected varies depending on the classification model and the execution algorithm, a detailed pre-exploration process is necessary. In the DNN model used in this study, we injected a fault by setting the parameters to the point at which the most misclassifications occurred (a width of 4000 and an offset of 2700).

**Figure 9:**
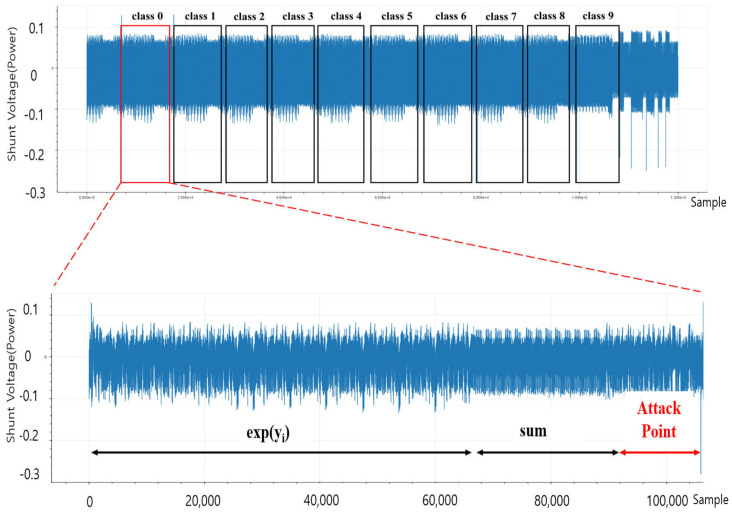
Reference waveform selection (92,000∼106,000 samples) for skipping the iterative instruction.

In order to generate a trigger using the SAD algorithm, it is necessary to obtain a reference waveform for the window of interest of the target device. The power consumption from the target board when executing the Softmax algorithm is shown in the upper part of Figure 9. In this figure, the x-axis represents the sample index, and the y-axis represents the normalized voltage measured across the shunt resistor. Additionally, since the target chip STM32F303 was set to allow for four samplings within one clock time (135.68 ns), the sample time interval of the power trace was 33.92 ns. The lower part of Figure 9 is the operation corresponding to class 0, and shows where the attack point for skipping the iterative instruction was implemented to execute the SAD algorithm right before the operation ends, generating a trigger when the SAD value exceeded a certain threshold.

**Figure 10:**
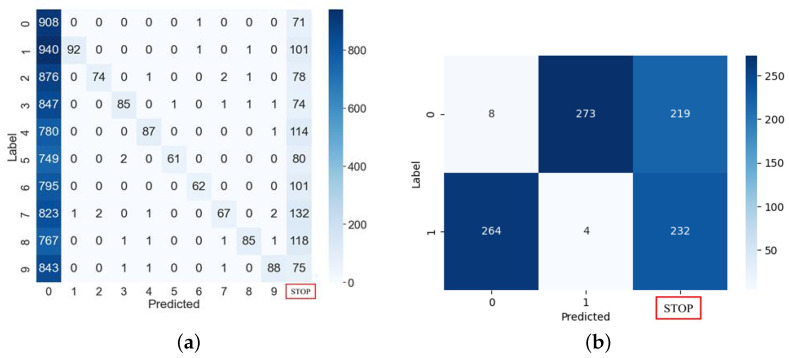
MLP classifications or stop in red box under glitch fault injection attacks. (**a**) Multi-classification. (**b**) Binary classification.

Figure 10a presents the results of the clock glitch fault attack on the MLP classification model using the MNIST (10 digits) test dataset comprising 10,000 samples. In this attack, most outputs were misclassified as 0, regardless of the input. We found that this occurred because the loop was skipped at the attack point in Algorithm 1. That is, if the branch statement at line 50 in Figure 4 is skipped, the attacker can intentionally decrease the output to zero through fault injection in the first iteration of Algorithm 1. Thus, this attack resulted in the misclassification of the samples as class 0. However, if a specific loop counter is skipped through fault injection, it will not be classified as a subsequent class. For example, if a loop statement is skipped when the loop counter value is 3, most inputs after class 3 will be misclassified.

The accuracy of the normal MLP model was 95.88%, and the accuracy of the attacked model decreased to 16.09%. The values in the rightmost column of the confusion matrix indicate where the device terminated irregularly due to the clock glitch. Excluding data in class 0, the accuracy of this fault attack was 7.77%. The result of the fault injection attack using the MNIST (two digits) test dataset comprising 1000 samples is shown in Figure 10b. Although the accuracy of the normal MLP model was 100%, the accuracy of the attacked model decreased to 1.2%.

Because the attack method in this approach targets only the Softmax function in the output layer, it is theoretically suitable for a range of applications, regardless of the DNN model or dataset used. In order to guarantee the universality and reliability of the fault injection attack, we conducted misclassification experiments on three CNN-based DNN models—InceptionNet, ResNet, and VGGNet—as well as MLP [34]. These CNN-based models are popularly used in image recognition because they can classify images with high probability. Therefore, we used the CIFAR-10 dataset for the multi-class classification that these models performed, and we used the Kaggle (Dogs vs. Cats) dataset for binary classification.

The results from the fault injection attacks on DNN models for multi-class classification are described in Table 2. Here, the CIFAR-10 dataset was tested only on three CNN-based DNN models considering their color image classification performance. Although the experimental results show that the MLP model achieved a normal classification accuracy of 95.88%, only 1069 samples were correctly classified out of 10,000 fault injection attempts. Specifically, while the VGGNet model, using 10,000 samples of the CIFAR-10 test dataset, achieved an accuracy of 93.30% under normal conditions, its accuracy decreased by up to 10% as a result of the fault injection attack.

The experimental results of binary classification are presented in Table 3. In the MLP model, the classification accuracy was 100% for the test datasets comprising 1000 samples, whereas only 12 samples were correctly classified after the fault injection attack. The Kaggle (Dogs vs. Cats) dataset consisting of 2000 images was tested on the three CNN-based DNN models. Although the experimental results indicate that normal classification accuracy from the InceptionNet model was 96.25%, it reduced to 1.1% as a result of the fault injection attack. The accuracy in binary classification is much lower than in multi-classification when a fault injection attack is launched. This is because the location of the fault injection is not in the loop instruction but in the input value, making it easy to determine the location of the fault injection accurately.

A comparison with other existing fault injection attacks is shown in Table 4. The practical fault attack using laser injection resulted in random misclassifications of more than 50% [24]. Khoshavi et al. found that glitches targeting clock signals can reduce accuracy by 20∼80% [35]. Liu et al. showed that an attacker can induce a misclassification rate of more than 98% in eight out of nine models using clock glitches [31]. A clock glitch fault injection into the Softmax function of a DNN can reduce accuracy to 11.2% [26].

Most of the previous studies were clock glitch fault attacks, and the recent experimental results showed that the classification accuracy has significantly decreased. We also performed misclassification attacks using clock glitches against Softmax and Sigmoid functions and experimentally confirmed that we can misclassify input images, which allowed us to reduce the accuracy by up to 16.09% in multi-classification and by up to 1.2% in binary classification.

## 5. Countermeasure

### 5.1. Proposed Activation Algorithm

The core of the fault injection attack on the Softmax function lies in artificially skipping the loop process during execution. Fukuda et al. proposed a countermeasure to defend against fault injection attacks on the Softmax algorithm [26]. This method uses random number initialization on the summation register. Since the countermeasure is to disrupt the pattern of the power waveform, it is vulnerable to the white-box attack scenario assumed in this paper.

The main ideas of our countermeasure are based on two factors. One is that we should verify the number of loops that occur during the execution of Softmax. To address this, the Softmax algorithm is designed to check whether the loop process has been executed completely or not. If you have not executed enough “For” loops for the number of output classes, you stop the process.

Even if a “For” loop executes normally, an injected fault can change the value of a variable in memory. Therefore, the other idea in the countermeasure is to check that the sum of output-normalized probability values is always 1.

(5) $$1=\Sigma_{i=0}^{J-1}Softmax\left(y_{i}\right)$$

This validation formula is based on Equation (1) and is a unique characteristic of the Softmax function. The idea in the second method is to check the memory value to prove that the operation inside the loop was performed properly.

The countermeasure at the algorithmic level to resist fault attacks is outlined in Algorithm 3. In the proposed countermeasure, we verify that the loop process is executed correctly through three conditional statements in the algorithm. The conditional statement in line 9 checks whether the first loop is executed fully, and line 16 verifies the second loop. Finally, line 19 confirms that the sum of the output values is equal to 1. Here, we emphasize that line 10 of Algorithm 1 and line 13 of Algorithm 3 must be computed differently. The reason is that, if line 13 of Algorithm 3 is calculated as $O_{k}=O_{k}/sum$, then the fault injection attack attempted at line 6 cannot be detected.

Similarly, a mathematical verification technique can be applied to detect a fault injected into the Sigmoid algorithm, which is an activation function used in the output layer of a binary classification model. The inverse function of output value *y* from the Sigmoid function shown in Equation (2) is as follows:

(6) $$x=log(\frac{y}{1-y})$$

An algorithmic countermeasure verifies whether the output is generated normally without faults by checking this condition. By adding this checking process to the final output stage of the model, we can defeat misclassification attacks. The proposed countermeasures to protect the Softmax and Sigmoid functions are logically sound. Their implementation requires almost no additional memory for storing a source code or RAM for computation. However, we found that the execution time of each function increased by slightly less than double, i.e., 88.72% for the Softmax function and 88.55% for the Sigmoid function. The results indicate that the countermeasure in the Softmax function added the exp() operation and an additional operation in the repeated loop. In the Sigmoid function, the log() operation was added. Nevertheless, the computational cost of these two functions is negligible in the context of the overall model execution time and thus does not affect their applicability in embedded systems in practice.
**Algorithm 3** The proposed Softmax function.**Require:** 
$y(y\in \left\{y_{0},\ldots ,y_{J-1}\right\})$
**Ensure:** 
$O(O\in \left\{O_{0},\ldots ,O_{J-1}\right\})$
  1:**for** (k=0;k<J;k++) **do**  2:    $O_{k}=0$  3:**end for**  4:sum=0,check=0  5:**for** (k=0;k<J;k++) **do**  6:    $O_{k}=exp\left(y_{k}\right)$  7:    $sum=sum+O_{k}$  8:**end for**  9:**if** (k!=J) **then**10:    exit(1);11:**end if**12:**for** (k=0;k<J;k++) **do**13:    $O_{k}=exp\left(y_{k}\right)/sum$14:    $check=check+O_{k}$15:**end for**16:**if** (k!=J) **then**17:    exit(1);18:**end if**19:**if** (check!=1) **then**20:    exit(1);21:**end if**


### 5.2. Fault Attacks on the Proposed Algorithm

To validate the proposed countermeasure algorithm, we conducted experiments on four DNN models using the same test dataset. In all cases where fault injection attacks were not launched on the Softmax and Sigmoid algorithm with countermeasures, image classification had the same accuracy as normal. Next, a fault injection attack was launched against an algorithm adopting the countermeasure, and the results confirmed that almost all faults were detected in the target device and device operation stopped.

**Figure 11:**
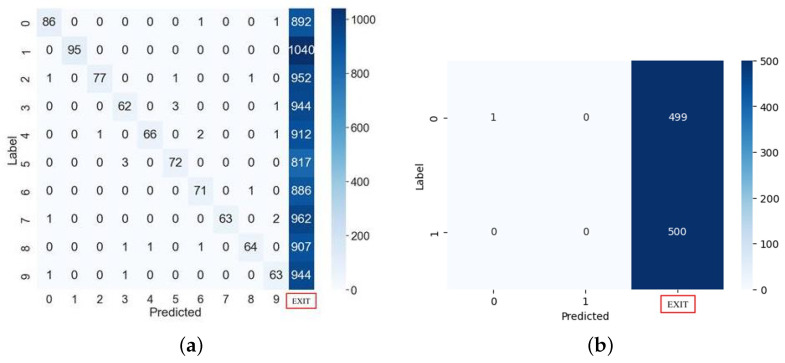
Misclassification or stop by fault attacks on MLP with the countermeasure. (**a**) Multi-classification. (**b**) Binary classification.

Figure 11a shows the results obtained from injecting a clock fault into the Softmax algorithm with countermeasures, and Figure 11b shows the results obtained from injecting the fault into the Sigmoid algorithm with countermeasures. In experiments using 10,000 test images on a multi-classification MLP with the countermeasure, we detected 9256 faults injected into the target chip. When the fault injection attack failed, we correctly classified 7.19% of the total images. The remaining 0.25% were misclassified, which was affected by the fundamental classification performance of the model. As shown in Figure 11b, when faults are injected into the Sigmoid function with the countermeasure, they can be detected with 100% probability. From the experiments on the microcontrollers implemented with the proposed countermeasure, we confirmed that it can completely prevent misclassification in MNIST digit images.

Our countermeasure only adds the operation to check whether the loop executes normally and to check the operations in lines 13 and 14 of Algorithm 3. Compared to the total execution time of the entire DNN model, this computational overhead is negligible. Therefore, our countermeasure can provide physical security of DNN models for classification in low-power and resource-constrained IoT devices. We applied the countermeasure to other CNN-based DNN models, obtained similar numerical results, and confirmed that the device stopped operating after detecting that a fault had been injected.

## 6. Conclusions

Deep learning, which has recently been attracting attention, is widely used for facial recognition and image detection in IoT-edge devices and smart homes. However, remote edge devices that run a DNN are exposed to threats such as fault injection attacks and side-channel attacks. In particular, glitch-fault injection attacks can easily lead to the misclassification of images through the utilization of simple tools.

The present paper has demonstrated that some clock glitch injection attacks can effectively induce faults in image-classification DNN models. The targets for fault injection attacks are the Softmax and Sigmoid functions at the output layer of multi-classification and binary classification, respectively.

To verify the applicability of fault injection attacks, we used the MNIST datasets for image classification and implemented DNN models on a 32-bit STM32F303 microcontroller. We designed an experimental environment to generate attack triggers external to the device using a SAD-based pattern-matching algorithm, considering realistic scenarios of fault injection attacks. By terminating the first loop of the Softmax function using glitches, the misclassification of class 0 data was easily induced.

The experimental results showed that the accuracy of multi-classification through MLP decreased from 95.88% to 16.09% following a clock glitch fault attack and decreased from 100% to 1.20% in the case of binary classification. Fault injection attacks on CNN-based multi-classification models resulted in an accuracy decrease of up to 10%. Furthermore, we proposed a countermeasure to glitch attacks on the Softmax function, which is based on checking the loop counter and validating computational correctness. And for the Sigmoid function, we applied a method to check whether the output is the same as the input by executing the inverse function on the output. In experiments injecting faults into a multi-classification MLP, our countermeasure was able to detect faults with a probability of up to 92.56%.

Consequently, we confirmed that our proposed Softmax and Sigmoid algorithms can sufficiently defeat fault injection attacks. Since our experiments were performed only on the STM32F303 microcontroller, further studies are needed to generalize our results. In particular, fault injection attacks by a laser or an EM other than clock glitch should be widely studied for hardware platforms with different design structures, such as RISC-V, x86, and FPGA architecture microcontrollers.

## Figures and Tables

**Table 1:** Structure of the MLP model for classification of the MNIST datasets.

Layer	Number of Neurons (Binary)	Activation Function (Binary)
Input layer	784	-
Hidden layer-1	64	ReLU
Hidden layer-2	32	ReLU
Output layer	10 (1)	Softmax (Sigmoid)

**Table 2:** Fault injection attacks on MLP and other CNN-based DNN models for multi-classification.

	Dataset	Accuracy	
		Normal	Attack
MLP	MNIST (10 digits, 0∼9)	95.88%	16.09%
InceptionNet	CIFAR-10	91.66%	10.61%
ResNet	CIFAR-10	88.38%	10.92%
VGGNet	CIFAR-10	93.30%	10.00%

**Table 3:** Fault injection attacks on MLP and other CNN-based DNN models for binary classification.

	Dataset	Accuracy	
		Normal	Attack
MLP	MNIST (2 digits, 0 vs. 1)	100%	1.20%
InceptionNet	Kaggle (Dogs vs. Cats)	96.25%	1.10%
ResNet	Kaggle (Dogs vs. Cats)	91.00%	4.40%
VGGNet	Kaggle (Dogs vs. Cats)	90.45%	1.75%

**Table 4:** Comparison of fault injection attacks.

	Target	Method	Goal	Effect
Bereir et al. [24]	Activation	Laser	Random	≥50%
	functions	injection	misclassification	Misclassification
Khoshavi et al. [35]	Clock	Clock glitch	Accuracy	20%∼80% accuracy
	signal		degradation	degradation
Liu et al. [31]	Clock	Clock glitch	Misclassification	≥98%
	signal			Misclassification
Fukuda et al. [26]	Softmax	Clock glitch	Misclassification	11.2%
	function		to class 0	Accuracy
Ours	Softmax/Sigmoid	Clock glitch	Misclassification	16.09%/1.2%
	function		to class 0 or others	Accuracy

## Data Availability

No new data were created or analyzed in this study. Data sharing is not applicable to this article.

## Abbreviations

The following abbreviations are used in this manuscript:

CNN	convolution neural network
DNN	deep neural network
EM	electromagnetic
FPGA	field-programmable gate array
IoT	Internet of Things
MLP	multilayer perceptron
